# Development and Validation of a Novel Score for Predicting Paroxysmal Atrial Fibrillation in Acute Ischemic Stroke

**DOI:** 10.3390/ijerph19127277

**Published:** 2022-06-14

**Authors:** Jiann-Der Lee, Ya-Wen Kuo, Chuan-Pin Lee, Yen-Chu Huang, Meng Lee, Tsong-Hai Lee

**Affiliations:** 1 Department of Neurology, Chiayi Chang Gung Memorial Hospital, No. 6, West Sec., Jiapu Road, Puzi City 613, Taiwan; jdlee540908@gmail.com (J.-D.L.); deepblue@cgmh.org.tw (Y.-C.H.); menglee5126@gmail.com (M.L.); 2 College of Medicine, Chang Gung University, No. 259, Wenhua 1st Rd., Guishan Dist., Taoyuan 333, Taiwan; thlee@adm.cgmh.org.tw; 3 Department of Nursing, College of Nursing, Chang Gung University of Science and Technology, No. 2, Sec. W., Jiapu Rd., Puzi City 613, Taiwan; 4 Health Information and Epidemiology Laboratory, Chang Gung Memorial Hospital, Chiayi 613, Taiwan; cblee@cgmh.org.tw; 5 Department of Neurology, Linkou Chang Gung Memorial Hospital, Taoyuan 333, Taiwan

**Keywords:** ischemic stroke, atrial fibrillation, heart rate, risk

## Abstract

Atrial fibrillation (AF)—whether paroxysmal or sustained—increases the risk of stroke. We developed and validated a risk score for identifying patients at risk of paroxysmal atrial fibrillation (pAF) after acute ischemic stroke (AIS). A total of 6033 patients with AIS who received 24 h Holter monitoring were identified in the Chang Gung Research Database. Among the identified patients, 5290 with pAF and without AF were included in the multivariable logistic regression analysis to develop the pAF prediction model. The ABCD-SD score (**A**ge, Systolic **B**lood pressure, **C**oronary artery disease, **D**yslipidemia, and **S**tandard **D**eviation of heart rate) comprises age (+2 points for every 10 years), systolic blood pressure (−1 point for every 20 mmHg), coronary artery disease (+2 points), dyslipidemia (−2 points), and standard deviation of heart rate (+2 points for every 3 beats per minute). Overall, 5.2% (274/5290) of patients had pAF. The pAF risk ranged from 0.8% (ABCD-SD score ≤ 7) to 18.3% (ABCD-SD score ≥ 15). The model achieved an area under the receiver operating characteristic curve (AUROCC) of 0.767 in the model development group. The ABCD-SD score could aid clinicians in identifying patients with AIS at risk of pAF for advanced cardiac monitoring.

## 1. Introduction

Research has indicated that patients with atrial fibrillation (AF) have an increased risk of acute ischemic stroke (AIS) [1]. The risk of recurrent stroke was reported to be 22% after an index cardioembolic stroke [2]. Jonas et al. reported that AF could be first diagnosed at or shortly after the index stroke in only approximately 20% of patients with AF-related stroke [3], and the increased risk of stroke was reported to be the same for paroxysmal AF (pAF) and permanent AF [4]. Several scoring systems have been developed to predict the risk of AF after an ischemic stroke. However, they mainly include echocardiographic measurements and laboratory parameters [5,6,7,8,9], which are not available for most patients with AIS in a stroke-unit diagnostic workup or daily practice. Due to the challenging and resource-intensive diagnosis, pAF is often underdiagnosed. Therefore, a simple method for identifying patients at high risk of incident pAF after AIS is needed.

In this study, data on clinical characteristics and initial in-hospital vital signs were utilized to develop and validate a scoring system for detecting pAF after AIS in a stroke-unit diagnostic workup. The AS5F score is an established risk score based on age and stroke severity (age: 0.76 points/year, stroke severity: National Institutes of Health Stroke Scale [NIHSS] score ≤ 5 = 9 points and >5 = 21 points) that can be used by clinicians to increase the diagnostic yield of pAF after ischemic stroke or transient ischemic attack. We hypothesized that the prediction accuracy of the AS5F score can be further improved by adding other clinical or laboratory parameters. Therefore, the performance of the score developed in this study was compared with that of the AS5F score.

## 2. Materials and Methods

### 2.1. Study Sample

The present study retrieved all data from the Chang Gung Research Database [10], the largest multi-institutional electronic medical record collection in Taiwan. We included patients who (1) were ≥ 18 years old, and (2) were diagnosed as having AIS (*International Classification of Diseases, 9th Revision, Clinical Modification* [*ICD-9-CM*] codes: 433.01, 433.11, 433.21, 433.31, 433.81, 433.91, 434.01, 434.11, and 434.91; *International Classification of Diseases, 10th Revision, Clinical Modification* [*ICD-10-CM*] code: I63) in their first 2 discharge diagnoses [11,12] between January 2010 and September 2018, (3) were admitted to the 7 hospitals of the Chang Gung health care system for at least 3 days, and (4) received 24 h Holter monitoring. All patients with stroke received a complete 12-lead ECG after admission. Most patients with stroke were admitted to general wards, and only those with severe stroke were admitted to the intensive care unit and monitored using 24 h bedside monitors. For patients admitted to the intensive care unit, if AF was suspected on the bedside monitor, a complete 12-lead electrocardiogram (ECG) and 24 h Holter monitoring were used to confirm the diagnosis of AF. Although a randomized trial revealed that longer heart monitoring in patients with cryptogenic stroke resulted in higher pAF detection rates [13], outcome improvement in patients with long-term rhythm monitoring has not been established. As several guidelines have recommended 24 h Holter monitoring after stroke [14,15,16], we enrolled AIS patients who received 24 h Holter monitoring in this study for further analysis.

AF was classified as pAF if the patient had (1) AF on a complete 12-lead ECG and AF was terminated within 7 days of onset [17] or (2) a self-terminating sequence of >30 s of irregular RR intervals and the presence of fibrillatory P waves on 24 h Holter ECG [18]; otherwise, it was classified as sustained AF. Key demographic and clinical characteristics were collected. *ICD-9-CM* and *ICD-10-CM* diagnosis codes were used to detect dyslipidemia (*ICD-9-CM* codes 272.0, 272.1, 272.2, 272.3, 272.4, and 272.9 and *ICD-10-CM* codes E78.0, E78.1, E78.2, E78.3, E78.4, and E78.5), coronary artery disease (CAD, *ICD-9-CM* codes 410, 411, 412, 413, and 414 and *ICD-10-CM* codes I20, I21, I22, I24, and I25), and congestive heart failure (*ICD-9-CM* codes 402.01, 402.11, 402.91, 404.01, 404.03, 404.11, 404.13, 404.91, 404.93, and 428 and *ICD-10-CM* codes I11.0, I13.0, I13.2, and I50) [19]. Vital sign data recorded in the acute stage (the first 3 days of hospitalization)–including systolic blood pressure, diastolic blood pressure, and heart rate (HR)–were obtained. The mean systolic blood pressure, diastolic blood pressure, and HR were derived from the recorded vital sign values. As AF is an abnormal heart rhythm characterized by rapid and irregular beating of the atrial chambers, we hypothesized that the variation in visit-to-visit HR would be higher in patients with pAF than in those without AF. The standard deviation of the HR (HR-SD) and the coefficient of variation of the HR (HR-CV) was calculated to represent the visit-to-visit HR variability in this study. As the Chang Gung Research Database does not include information on stroke severity, the following validated method was used to estimate stroke severity. Seven items from the inpatient claims for the index hospitalization were used to calculate the stroke severity index. The stroke severity index was then converted into an NIHSS score by using the following equation: estimated NIHSS (eNIHSS) score = 1.1722 × stroke severity index − 0.7533 [20]. This study was performed in accordance with the Helsinki Declaration. As only anonymous data were analyzed in this study, the requirement for informed consent was waived. The Ethics Committee of Chang Gung Memorial Hospital, Chiayi Branch, Taiwan, approved the study (202001990B0).

### 2.2. Statistical Analysis

Descriptive statistics are presented as numbers (percentages) for categorical data and medians (interquartile ranges) for continuous data. Differences between groups were tested using the Kruskal–Wallis rank test for continuous data and the chi-square test for categorical data.

To develop the prediction model for pAF, patients with pAF and without any AF were included in the analysis. The study population was randomly split into 2 groups using the following method. First, a set of random numbers from a uniform distribution between 0 and 1 was generated, with a random number for each patient. Next, the patients in the study population were sorted by these random numbers. The first two-thirds of patients on the list comprised the score development group, and the remaining third comprised the validation group. Multivariable logistic regression analysis with backward variable selection (probability for removal > 0.05) was performed to identify predictors of pAF. According to previous studies [21,22,23,24], the potential candidate predictors were age, sex, eNIHSS score, hypertension, diabetes mellitus, dyslipidemia, CAD, congestive heart failure, smoking status, prior stroke or transient ischemic attack, total cholesterol level, triglyceride level, creatinine level, alanine aminotransferase level, mean systolic blood pressure, mean diastolic blood pressure, mean HR, HR-CV, and HR-SD. Stroke severity was categorized into mild (eNIHSS score ≤ 5), moderate (eNIHSS score 6–13), and severe (eNIHSS score > 13), with mild severity as the reference group. Data are presented as adjusted odds ratios and 95% confidence intervals where appropriate.

### 2.3. Risk Score Development and Internal Validation

To facilitate the use of the prediction model, a simplified risk scoring system was created on the basis of the regression coefficients of the multivariable logistic regression model [25]. A total risk score was calculated by summing all the points corresponding to the predictors present in any given patient. After the score model had been established, it was validated using the validation group of patients. The discriminatory ability of the risk score was evaluated using the area under the receiver operating characteristic curve (AUROCC). Calibration was assessed by plotting the predicted risk versus the observed risk for quintiles of the predicted probability. For a model with favorable calibration, the data points on the calibration curve are located close to the plot’s 45° diagonal line.

For comparison, the AS5F score was calculated for the validation group. The AS5F score, based solely on age and NIHSS score, was devised to detect pAF after ischemic stroke [21]. Uphaus et al. found that the AS5F threshold of 67.5 points corresponded to the pAF detection rate of 5.2% (which was the lower limit of the high-risk group defined using AS5F); in other words, at this threshold, one patient with pAF was detected during 72-h Holter monitoring for every 20 patients screened [21]. AUROCCs were calculated to compare the models’ performance. In addition, the clinical usefulness and net benefit (i.e., the ability to make better decisions with a model than without) were estimated through decision curve analysis [26]. All statistical analyses were performed using SPSS (version 22.0; IBM, Armonk, NY, USA) and R (version 4.0).

## 3. Results

### 3.1. Baseline Characteristics

In total, 6033 patients were enrolled (Supplementary Figure S1), of whom 274 (4.5%) had pAF and 743 (12.3%) had sustained AF. For patients with pAF and those without AF, 109,822 vital sign measurements were obtained during the first 3 days of hospitalization. The median number of measurements per patient was 12 (interquartile range 9–18). Compared with the patients without AF, the pAF group was older and predominately female and had higher eNIHSS scores, a lower prevalence of smoking, lower baseline total cholesterol and triglycerides levels, lower mean systolic blood pressure and diastolic blood pressure, and higher HR-SDs and HR-CVs; in addition, fewer patients with pAF had dyslipidemia, and more had CAD and congestive heart failure (Table 1). We included 5290 patients with pAF and without AF in the multivariable logistic regression analysis to develop the pAF prediction model.

### 3.2. Score Development and Validation

**Figure 1:**
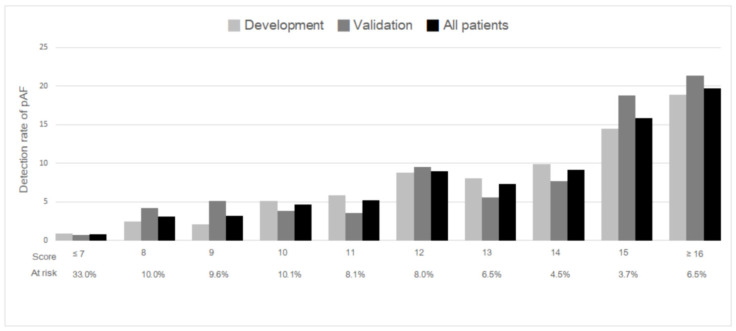
Rates of pAF detection across risk scores. “At-risk” is defined as the proportion of all patients with the respective score. Abbreviations: pAF, paroxysmal atrial fibrillation.

The multivariable logistic regression analysis in the development group revealed 5 risk factors independently associated with pAF (Table 2). Among them, age, CAD, and HR-SD were positive predictors, whereas dyslipidemia and mean systolic blood pressure were negative predictors. As the mean systolic blood pressure was significantly negatively associated with the risk of pAF in this study, we used the coefficient of the mean systolic blood pressure (per 20 mmHg) as the number of regression units to reflect a reduction of 1 point in the final scoring system. The points assigned to other significant predictors were obtained by dividing each coefficient by that of the mean systolic blood pressure and rounding to the nearest integer. Each patient’s score was derived by summing the points for each predictor; 2 points were added for every 10 years of age, 1 point was subtracted for every 20 mmHg of mean systolic blood pressure, 2 points were added for CAD, 2 points were subtracted for dyslipidemia, and 2 points were added for every 3 beats per minute of HR-SD. The final risk score was named the ABCD-SD score (**A**ge, Systolic **B**lood Pressure, **C**AD, **D**yslipidemia, and HR-**SD**). The total individual scores ranged between −3 and 26. For clinical practicality, we stratified the total risk score into 3 risk categories: low (≤7), medium (8–14), and high (≥15) risk. Figure 1 demonstrates the risk of pAF in the development group, validation group, and entire study population. In the entire study population, the overall risk of pAF was 5.2%, and the median total score was 9 points. Approximately 10.2% of patients had a score ≥ 15 points, corresponding to a pAF risk of 18.3%. By contrast, patients with a score of ≤7 points only had a 0.8% risk of pAF. The calibration plot (Supplemental Figure S2) illustrates that close agreement was observed between the predicted and observed probabilities, indicating that the risk score was well-calibrated. Table 3 provides the number of patients at risk and the risk of pAF across the 3 risk categories in the entire study population.

**Figure 2:**
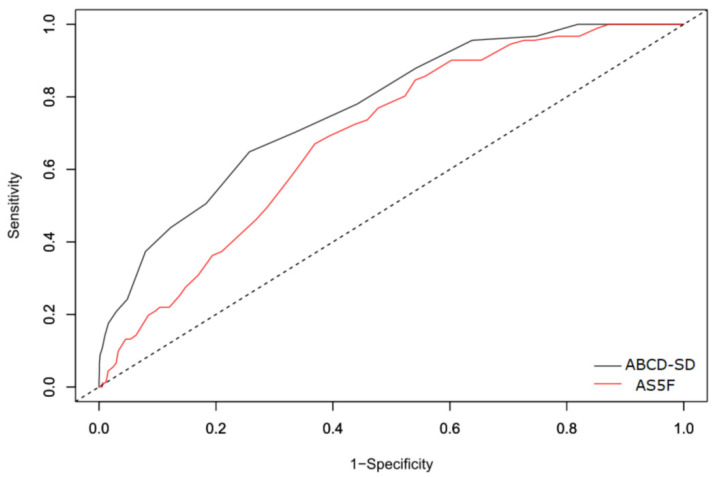
ABCD-SD score used for the prediction of paroxysmal atrial fibrillation in the validation group (AUROCC 0.769) compared with AS5F score (AUROCC 0.689, *p* = 0.022). Abbreviations: AUROCC, the area under the receiver operating characteristic curve.

**Figure 3:**
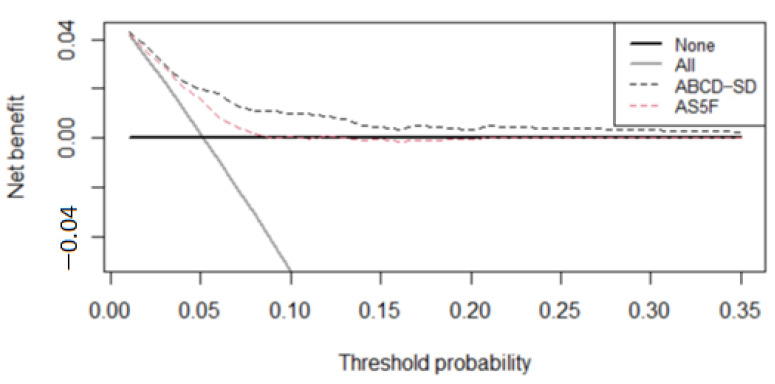
Decision curve analysis for the risk scores (ABCD-SD and AS5F scores). The diagonal gray line indicates the net benefit assuming all patients are at risk of paroxysmal atrial fibrillation, and the horizontal black line assumes no patients are at risk of paroxysmal atrial fibrillation.

The AUROCC of the ABCD-SD score for the score development group was 0.767 (95% confidence interval 0.736–0.798). In the validation group, the ABCD-SD score had a strong discriminative ability, with an AUROCC of 0.769 (95% confidence interval 0.724–0.815), which was significantly higher than that of the AS5F score (0.689, 95% confidence interval 0.642–0.737, *p* = 0.022; Figure 2). Figure 3 depicts the net benefit curves of the risk scores across risk thresholds. The ABCD-SD score demonstrated higher clinical usefulness than the AS5F score.

## 4. Discussion

We developed and validated a simple risk score, called the ABCD-SD score, for predicting pAF after AIS by using clinical characteristics and routine vital sign measurements. The ABCD-SD score demonstrates superior discrimination than the AS5F score in predicting pAF.

A number of risk scores have been developed to predict the risk of AF after stroke [27]. Among these risk scores, 8 risk scores are based on readily available variables and are considered suitable for routine clinical use, including AS5F, C_2_HEST, CHADS_2_, CHA_2_DS_2_-VASc, CHASE-LESS, HATCH, HAVOC, and Re-CHARGE-AF scores [21,22,23,28,29,30,31]. The predictive performance of these risk scores has been compared, and the CHASE-LESS and AS5F scores showed a better predictive performance than the other 6 risk scores [27]. Since both the AS5F and ABCD-SD scores were developed to predict the risk of pAF in a stroke-unit diagnostic workup, the AS5F score was adopted as the benchmark to assess the performance of the ABCD-SD score.

Several clinical variables were positively and significantly associated with pAF. As expected, age was a strong predictor of pAF [32]. Additionally, CAD was identified as a predictor, in line with the results of a non-stroke cohort in the Framingham Heart Study [33]. Low HR variability (measured as the variation in the beat-to-beat interval) is associated with a higher risk of AF [34], and the frequency of visit-to-visit HR variability is associated with the risk of new-onset AF in the general population [24]. However, few studies have investigated the correlation between visit-to-visit HR variability and AF in patients with AIS. AF is typically defined as irregular heartbeat and usually with an HR of 100–175 beats per minute; therefore, the mean HR and visit-to-visit HR variability are likely to be higher in patients with AF than in those without AF. In the current study, the visit-to-visit HR variability, expressed as HR-SD and HR-CV, was significantly greater in the patients with pAF and sustained AF than in the patients without AF (Table 1). In addition, HR-SD was an independent risk factor for pAF in the multivariable logistic regression analysis (Table 2). Consequently, the HR-SD was included in the ABCD-SD score as a novel and critical predictor of pAF risk.

Previously, a higher NIHSS score was identified as a predictor of pAF [35], and adding stroke severity to existing risk scores (e.g., the CHADS_2_ and CHA_2_DS_2_-VASc scores) improved their ability to predict newly diagnosed AF [36]. However, we did not include stroke severity in the ABCD-SD score. Nevertheless, the performance of the ABCD-SD score was superior to that of the AS5F score, which includes stroke severity in its scoring system (Figure 2 and Figure 3). In the current study, age, HR-SD, CAD, and dyslipidemia were associated with stroke severity in the development group (Supplementary Table S1). When the eNIHSS score was divided into 3 categories (mild, 0–5; moderate, 6–13; and severe, >13), a significant increase in age and HR-SD was observed across the groups (63.5 ± 13.0 years and 6.8 ± 2.8 beats per minutes in the mild group; 66.2 ± 15.0 years and 7.6 ± 3.2 beats per minute in the moderate group; and 69.3 ± 14.3 years and 9.2 ± 3.4 beats per minute in the severe group; *p* for trend for both groups, <0.001). In addition, the prevalence of CAD was significantly higher, and the prevalence of dyslipidemia was significantly lower with greater stroke severity (prevalence of CAD: 8.2% in the mild group, 10.9% in the moderate group, and 11.1% in the severe group, *p* = 0.032; prevalence of dyslipidemia: 52.0% in the mild group, 48.3% in the moderate group, and 41.3% in the severe group, *p* < 0.001; Supplementary Table S1). This implies that age, HR-SD, CAD, and dyslipidemia can serve as substitutes for stroke severity in predicting pAF risk.

The CHADS_2_ and CHA_2_DS_2_-VASc scores were originally developed to predict stroke in patients with AF and were later found to predict new-onset AF after ischemic stroke [23]. Unlike the CHADS_2_ and CHA_2_DS_2_-VASc scores, the ABCD-SD score has been used to assess the risk of pAF during hospitalization instead of new-onset AF after discharge. As oral anticoagulation is recommended in all patients with AF and a history of ischemic stroke [37], detection of AF in patients with stroke has immediate effects on treatment. For clinicians, identifying pAF during hospitalization may be more realistic than predicting future new-onset AF after discharge.

A unique characteristic of the ABCD-SD score is the inclusion of variables negatively associated with pAF, namely dyslipidemia and mean systolic blood pressure. Although hypercholesterolemia is a well-known risk factor for cardiovascular disease, it has not been thoroughly investigated as a risk factor for AF. Many large community-based cohort studies have observed inverse associations between blood lipid levels and AF incidence [38,39,40,41,42], and this observation is compatible with the results of the present study (Table 1 and Table 2). Although the exact mechanism underlying the inverse association between dyslipidemia and AF remains unclear, a phenomenon known as the “cholesterol paradox” seems to exist in AF [43]; several mechanisms have been proposed to explain this phenomenon [44,45,46,47]. Hypertension was previously identified as a risk factor for AF in the development of the CHADS_2_ and CHA_2_DS_2_-VASc scores [23], however, the current study observed that patients with pAF and sustained AF tended to have lower systolic blood pressure levels than those without AF after AIS (Table 1), which was compatible with the findings of other studies [48,49,50]. The lower systolic blood pressure in patients with AF in the early stage of AIS may be related to the pathogenetic mechanism underlying the specific stroke subtype but not necessarily to original blood pressure. Therefore, dyslipidemia and mean systolic blood pressure after AIS are considered to be predictors of lower pAF risk in the ABCD-SD score.

The detection of pAF in patients with AIS is challenging. Although extended and serial cardiac monitoring effectively increases pAF detection rates after transient ischemic attack or stroke [51], such strategies are resource-intensive and costly. Even for patients in whom the cause of stroke is unknown, only 19% of hospitals in high-income countries routinely perform prolonged (>24 h) cardiac monitoring [52]. Studies that did not use risk-stratification tools have demonstrated that routine 24 h Holter monitoring can detect pAF in only approximately 2% of patients with cerebral infarction [53,54]. In the current study, the number needed to screen and detect a case of pAF was 19 using 24 h Holter monitoring without risk-stratification tools (Table 3). Although only 10.2% of patients were categorized as high risk for pAF according to the ABCD-SD score, the number needed for screening was decreased to 5 in this category. Thus, 24 h Holter monitoring or long-term ECG monitoring can be reserved for patients with a high ABCD-SD score. By contrast, 33.2% of patients were categorized as low risk for pAF, and the number needed for screening was 125 in this category. As unselected ECG monitoring and additional workup procedures may lead to the unnecessary use of health care resources and even cause harm to patients [55], the potential of the ABCD-SD score to minimize the potential harms and maximize the efficiency of limited medical resources is promising. We strongly suggest 24 h Holter monitoring for patients with AIS but without AF at baseline and with ABCD-SD score ≥ 15 because this can increase the detection rate of pAF from 5.2% to 18.3% (Table 3).

Moreover, the ABCD-SD score has several advantages. First, the score can be determined rapidly within 3 days of hospital admission, thereby allowing the prioritization of high-risk patients for advanced cardiac monitoring for pAF detection. Second, this score is easily calculated at the bedside because the score was designed using an integer point system. All its components are readily available from patient history and routine vital sign measurements. By contrast, many other risk models require additional effort to obtain necessary variables, such as ECG features [56], echocardiographic parameters [8], and blood biomarkers [57], leading to greater consumption of health care resources.

This study has several limitations. First, pAF was diagnosed through 24 h Holter monitoring, which may have been insufficient to detect all instances of pAF, resulting in underdiagnosis. Second, the type of ischemic stroke was not included in the analysis because this information is not available in the Chang Gung Research Database. The predictors selected in the final model and the weight assigned to each predictor may vary across different types of ischemic stroke. Third, 24 h Holter monitoring was performed per the clinical judgment of the physician-in-charge, which may have caused some selection bias. Finally, we determined the mean systolic blood pressure and HR-SD from the measurements taken at the first 3 days and did not record these values at the same intervals during hospitalization; this may have underestimated or overestimated the associations of systolic blood pressure and HR-SD with pAF risk.

## 5. Conclusions

The ABCD-SD score is a simple risk score based on readily available clinical characteristics and routine vital sign measurements. In addition to the established clinical score (AS5F), the ABCD-SD score can identify patients with AIS at high pAF risk more effectively and help prioritize specific patients for advanced cardiac monitoring in real-world practice. Thus, the ABCD-SD score may help overcome the obstacle of limited resources.

## Figures and Tables

**Table 1:** Baseline characteristics of the study population.

	Acute Ischemic Stroke				*p* Value
	Total (*N* = 6033)	Without AF (*N* = 5016)	With pAF (*N* = 274)	With Sustained AF (*N* = 743)	
Age, years	67 (57–76)	65 (55–75)	74 (65–81) *	74 (65–81) **	<0.001
Male	3921 (65.0)	3342 (66.6)	157 (57.3) *	422 (56.8) **	<0.001
eNIHSS	4 (4–9)	4 (4–7)	4 (4−11) *	4 (4–11) **	<0.001
eNIHSS ≤ 5	4009 (66.5)	3511 (70.0)	153 (55.8)	345 (46.4)	
eNIHSS 6–13	1151 (19.1)	929 (18.5)	58 (21.2)	164 (22.1)	
eNIHSS > 13	873 (14.5)	576 (11.5)	63 (23.0)	234 (31.5)	
Hypertension	4391 (72.8)	3663 (73.0)	206 (75.2)	522 (70.3)	0.188
Diabetes mellitus	2169 (36.0)	1874 (37.4)	83 (30.3)	212 (28.5) **	<0.001
Dyslipidemia	2956 (49.0)	2595 (51.7)	92 (33.6) *	269 (36.2) **	<0.001
Congestive heart failure	334 (5.5)	212 (4.2)	27 (9.9) *	95 (12.8) **	<0.001
Coronary artery disease	578 (9.6)	445 (8.9)	40 (14.6) *	93 (12.5) **	< 0.001
Current smoker	1839 (30.5)	1631 (32.5)	65 (23.7) *	143 (19.2) **	< 0.001
Prior stroke or TIA	1280 (21.2)	1058 (21.1)	53 (19.3)	169 (22.7)	0.436
Total cholesterol, mmol/L	4.53 (3.91–5.23)	4.58 (3.96–5.31)	4.30 (3.76–4.92) *	4.30 (3.68–4.92) **	<0.001
Triglyceride, mmol/L	1.24 (0.89–1.79)	1.31 (0.94–1.86)	1.04 (0.79–1.54) *	0.96 (0.70–1.32) **	<0.001
Creatinine, μmol/L	84.86 (68.95–107.85)	83.98 (68.95–106.96)	89.28 (72.49–113.15)	88.40 (70.72–112.27) **	0.002
ALT, U/L	21 (16–29)	21 (16–29)	20 (15–27)	20 (15–28) **	0.006
Mean SBP, mmHg	148.3 (135.4–162.8)	149.4 (136.4–164.1)	144.6 (132.5–158.5) *	142.1 (132.6–155.8) **	<0.001
Mean DBP, mmHg	83.5 (76.8–91.1)	84.0 (77.1–91.8)	79.8 (73.9–87.2) *	81.8 (75.5–89.2) **	<0.001
Mean HR, bpm	73.4 (66.4–80.3)	72.7 (65.9–79.4)	73.8 (66.2–81.1)	77.8 (70.2–87.8) **	<0.001
SD of HR, bpm	6.9 (5.1–9.3)	6.6 (5.0–8.6)	8.6 (6.2–12.4) *	9.5 (7.2–12.4) **	<0.001
CV of HR	0.09 (0.07–0.12)	0.09 (0.07–0.12)	0.12 (0.09–0.16) *	0.12 (0.09–0.15) **	<0.001

Data are presented as numbers (percentages) for categorical data and medians (interquartile ranges) for continuous data. Abbreviations: AF, atrial fibrillation; pAF, paroxysmal atrial fibrillation; eNIHSS, estimated National Institute of Health Stroke Scale; TIA, transient ischemic attack; ALT, alanine aminotransferase; SBP, systolic blood pressure; DBP, diastolic blood pressure; HR, heart rate; bpm, beats per minute; SD, standard deviation; CV, coefficient of variation.* Significant difference between patients with pAF and without AF by post hoc analysis. ** Significant difference between patients with sustained AF and without AF by post hoc analysis.

**Table 2:** Logistic regression model for diagnosis of paroxysmal atrial fibrillation.

	β-Coefficient	OR	95% CI	*p* Value	Points
Age (per 10 years)	0.522	1.69	1.48–1.92	<0.001	2
Coronary artery disease	0.576	1.78	1.16–2.72	0.008	2
Dyslipidemia	−0.513	0.60	0.43–0.83	0.002	−2
SD of heart rate (per 3 bpm)	0.439	1.55	1.38–1.75	<0.001	2
Mean SBP (per 20 mmHg)	−0.240	0.79	0.67–0.93	0.005	−1

Abbreviation: OR, odds ratio; CI, confidence interval; SD, standard deviation; bpm, beats per minute; SBP, systolic blood pressure.

**Table 3:** Risk categories and corresponding detection rate of paroxysmal atrial fibrillation.

Risk Score	Risk Category	N (%)	Detection Rate of pAF
≤7	Low	1757 (33.2)	0.8%
8–14	Medium	2992 (56.6)	5.5%
≥15	High	541 (10.2)	18.3%
Overall		5290 (100)	5.2%

Abbreviation: N, number; pAF, paroxysmal atrial fibrillation.

## Data Availability

The data supporting the findings of the article is available in the Chang Gung Research Databank at Chang Gung Memorial Hospital, Chiayi Branch. These data can be available after obtaining approval from our local IRB.

## Supplementary Materials

The following supporting information can be downloaded at: https://www.mdpi.com/article/10.3390/ijerph19127277/s1, Figure S1: Flow chart of patient selection; Figure S2: Calibration plot of the validation cohort; Table S1: Characteristics of the age, HR-SD, CAD and dyslipidemia according to eNIHSS score subgroups.
