# Supplementary material for: Development and Validation of a Novel Score for Predicting Paroxysmal Atrial Fibrillation in Acute Ischemic Stroke
Source: Int J Environ Res Public Health. 2022 Jun 14;19(12):7277. doi: 10.3390/ijerph19127277 (PMC9223581; doi:10.3390/ijerph19127277)
Supplement: Supplementary file 1 [file ijerph-19-07277-s001.zip › Supplementary Table S1.pdf]

Supplementary Table S1. Characteristics of the age, HR-SD, CAD and dyslipidemia according to eNIHSS score subgroups

	**Stroke severity**			
	**eNIHSS 0–5 (N = 2415)**	**eNIHSS 6–13 (N = 662)**	**eNIHSS > 13 (N = 450)**	***p*-value**
Age, years	63.5 (13.0)	66.2 (15.0)	69.3 (14.3)	< 0.001
HR-SD, bpm	6.8 (2.8)	7.6 (3.2)	9.2 (3.4)	< 0.001
CAD	199 (8.2)	72 (10.9)	50 (11.1)	0.032
Dyslipidemia	1256 (52.0)	320 (48.3)	186 (41.3)	< 0.001

Data are n (%) for categorical data and mean (standard deviation) for continuous data.

Abbreviations: HR-SD, standard deviation of heart rate; CAD, coronary artery disease; eNIHSS, estimated National Institutes of Health Stroke Scale; bpm, beats per minute.
