# Supplementary figures and images for: Development and Validation of a Novel Score for Predicting Paroxysmal Atrial Fibrillation in Acute Ischemic Stroke
Source: Int J Environ Res Public Health. 2022 Jun 14;19(12):7277. doi: 10.3390/ijerph19127277 (PMC9223581; doi:10.3390/ijerph19127277)

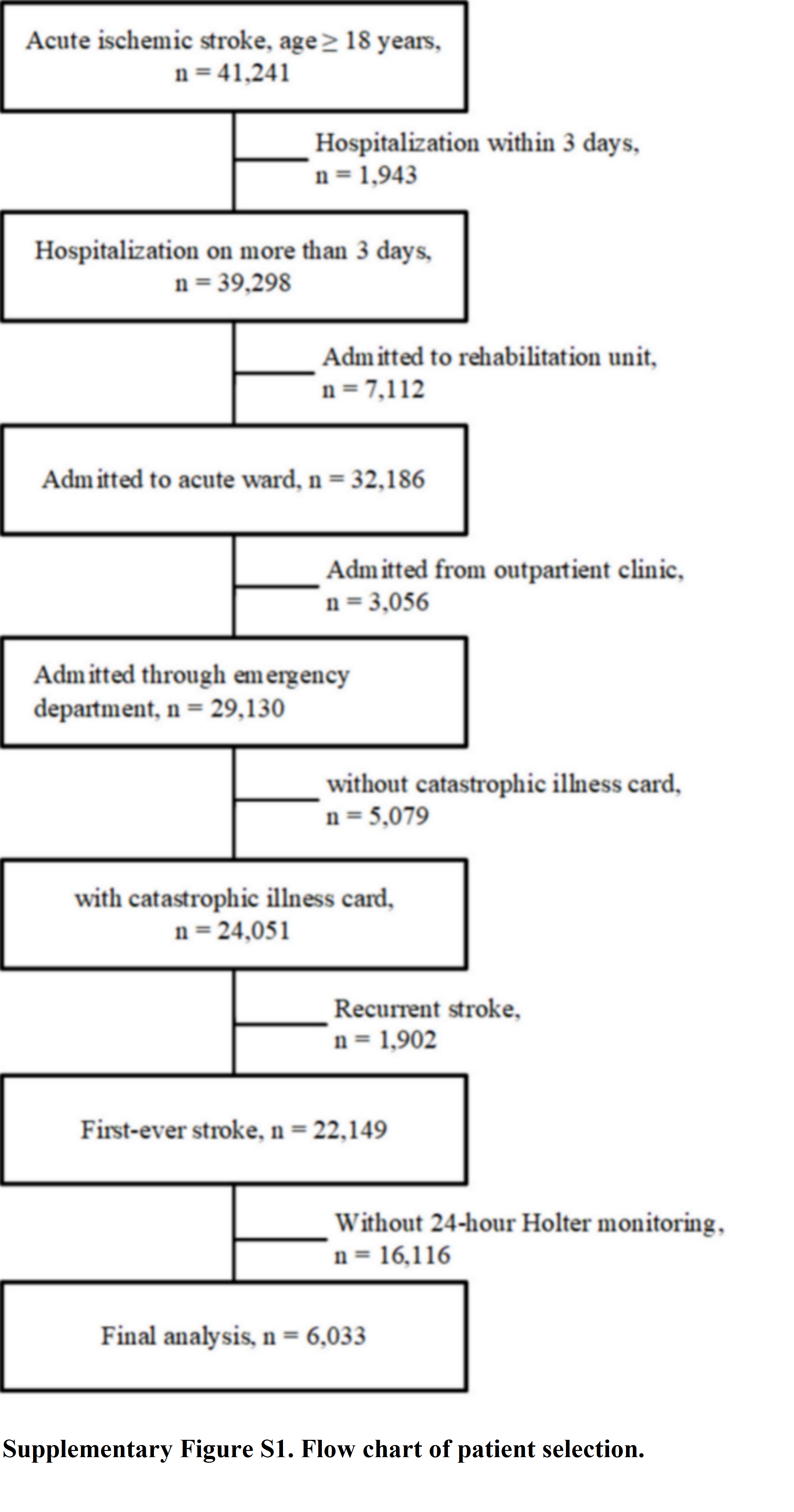

Supplement: Supplementary file 1 [file ijerph-19-07277-s001.zip › Supplementary Figure S1.tif]

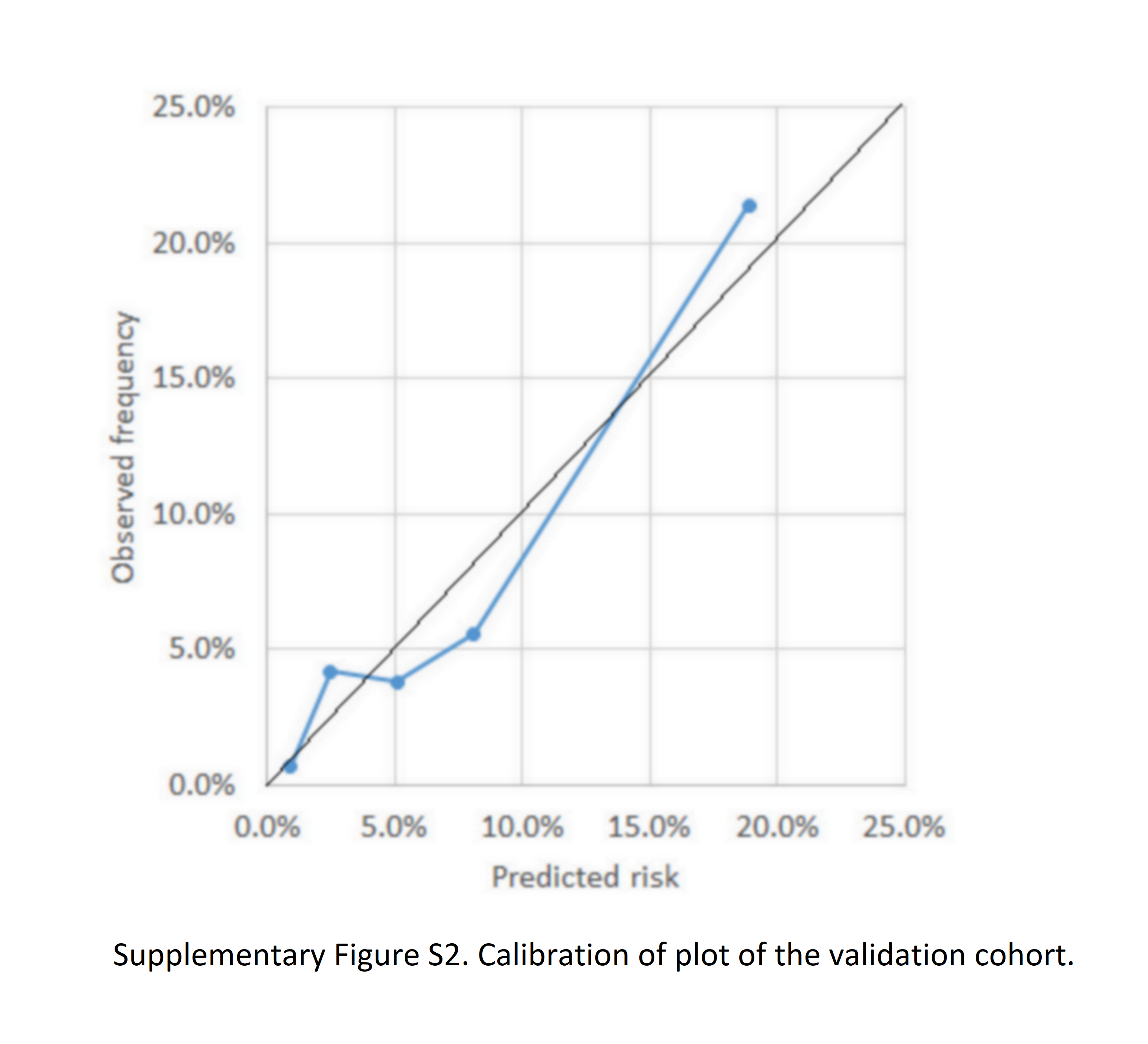

Supplement: Supplementary file 1 [file ijerph-19-07277-s001.zip › Supplementary Figure S2.tif]
